# LPCAT1 enhances the invasion and migration in gastric cancer: Based on computational biology methods and in vitro experiments

**DOI:** 10.1002/cam4.5991

**Published:** 2023-05-15

**Authors:** Zu‐Xuan Chen, Liang Liang, He‐Qing Huang, Jian‐Di Li, Rong‐Quan He, Zhi‐Guang Huang, Rui Song, Gang Chen, Jian‐Jun Li, Zheng‐Wen Cai, Jie‐An Huang

**Affiliations:** ^1^ Department of Medical Oncology The Second Affiliated Hospital of Guangxi Medical University Nanning People's Republic of China; ^2^ Department of General Surgery The Second Affiliated Hospital of Guangxi Medical University Nanning People's Republic of China; ^3^ Department of Radiotherapy The First Affiliated Hospital of Guangxi Medical University Nanning People's Republic of China; ^4^ Department of Pathology The First Affiliated Hospital of Guangxi Medical University Nanning People's Republic of China; ^5^ Department of Medical Oncology The First Affiliated Hospital of Guangxi Medical University Nanning People's Republic of China; ^6^ Department of Gastroenterology The Second Affiliated Hospital of Guangxi Medical University Nanning People's Republic of China

**Keywords:** computational biology, gastric cancer, immunohistochemistry, in vitro experiments, lysophosphatidylcholine acyltransferase 1 (LPCAT1), standard mean difference (SMD)

## Abstract

**Background and Aim:**

The biological functions and clinical implications of lysophosphatidylcholine acyltransferase 1 (LPCAT1) remain unclarified in gastric cancer (GC). The aim of the current study was to explore the possible clinicopathological significance of LPCAT1 and its perspective mechanism in GC tissues.

**Materials and Methods:**

The protein expression and mRNA levels of LPCAT1 were detected from in‐house immunohistochemistry and public high‐throughput RNA arrays and RNA sequencing. To have a comprehensive understanding of the clinical value of LPCAT1 in GC, all enrolled data were integrated to calculate the expression difference and standard mean difference (SMD). The biological mechanism of LPCAT1 in GC was confirmed by computational biology and in vitro experiments. Migration and invasion assays were also conducted to confirm the effect of LPCAT1 in GC.

**Results:**

Both protein and mRNA expression levels of LPCAT1 in GC were remarkably higher than those in noncancerous controls. Comprehensively, the SMD of LPCAT1 mRNA was 1.11 (95% CI = 0.86–1.36) in GC, and the summarized AUC was 0.85 based on 15 datasets containing 1727 cases of GC and 940 cases of non‐GC controls. Moreover, LPCAT1 could accelerate the invasion and migration of GC by boosting the neutrophil degranulation pathway and disturbing the immune microenvironment.

**Conclusion:**

An increased level of LPCAT1 may promote the progression of GC.

## 1 INTRODUCTION

Gastric cancer (GC) is a major malignancy of the digestive system.^1^ In the past few decades, the global morbidity rate and incidence rate of GC have declined, partly due to the enhanced sanitary standard, the improvement of food preservation, the sufficient intake of fresh fruits and vegetables, and the eradication of *Helicobacter pylori*.^2^, ^3^ However, new cases of GC exceeded 1 million in 2018, and approximately 780,000 patients died, according to the latest official statistics issued by the World Health Organization. Globally, the incidence rate in males was double that in females. The high‐risk areas of this cancer included East Asia, East Europe, and Central and South America, whereas South Asia, North and East Africa, North America, Australia, and New Zealand were labeled as low‐risk areas. The latest statistics from the American Cancer Society showed that approximately 26,380 new cases of GC are projected to occur in 2022, of which 11,090 cases are likely to result in death.^4^ In terms of treatment technology, although neoadjuvant chemoradiotherapy and targeting therapy are believed to be effective, surgery has proven to be the sole radical treatment.^5^, ^6^, ^7^, ^8^ Contemporarily, the survival rate of patients with early GC was more than 95% owing to surgical improvements, the development of chemotherapy and radiotherapy, and the practice of neoadjuvant treatments. Nevertheless, the difficulty with early detection has led to the fact that the majority of patients have been diagnosed at late stages and have thus failed to undergo optimal surgical treatment. In addition, patients with metastatic GC have poor prognoses.^9^, ^10^, ^11^, ^12^ The current situation can be ameliorated on the condition that we clarify the molecular mechanism of the onset and metastasis of GC.

Molecules related to metabolism play a pivotal part in GC. Lysophosphatidylcholine acyltransferase 1 (LPCAT1), also known as AYTL2, is a type of lipid metabolism‐associated gene situated at 5p15.33 of chromosome 5 with a length of 69,555 bp.^13^ LPCAT1 is reported to take part in various cancers. In prostatic cancer, the expression of LPCAT1 has been positively correlated with the progression of prostatic cancer, and its protein level has also been positively associated with the grading and staging of prostatic cancer.^14^ In breast cancer, the expression of LPCAT1 has remained high and has been positively linked with the progression of cancer.^15^ Moreover, aberrantly high expression of LPCAT1 has been examined in some malignant tumors, such as endometrial cancer, esophageal squamous cell carcinoma, hepatic carcinoma, and lung carcinoma.^13^, ^16^, ^17^, ^18^, ^19^ Currently, only one study published in 2016 indicated that LPCAT1 exhibited elevated expression in GC based on a small sample^20^ but only focused on the different expression between tumor and non‐tumor tissue. A comprehensive analysis with a large sample size is needed, and the clinical significance and molecular mechanism of LPCAT1 in GC needs to be further determined.

In the current study, we took advantage of gene chips, RNA‐seq data, ChIP‐seq data, single‐cell RNA‐seq data, in vitro experiments, and an immunohistochemistry (IHC) test on tissue microarrays to further investigate the clinical significance and molecular mechanism of LPCAT1 in GC.

## 2 MATERIALS AND METHODS

### 2.1 Expression of LPCAT1 protein in gastric cancer and noncancerous tissues

#### 2.1.1 Immunohistochemistry test of LPCAT1

Our study was approved by the medical ethics committee of Second Affiliated Hospital of Guangxi Medical University and Fanpu Biotech, lnc. Guilin Guangxi. All the patients had completed the informed consent form.

To preliminary explore the expression of LPCAT1 protein in GC, a total of 179 cases of GC and 147 noncancerous cases were contained in the tissue microarray purchased from Pantomics, Inc. (Richmond). From the microarray, the researchers acquired important clinical data that included age, sex, pathological grade of tumors, and clinical stage. The polyclonal antibody of LPCAT1 (1:100 dilution; catalog no. ab214034) was provided by Abcam Biotechnology, Inc., Cambridge, and the staining results were independently evaluated by two pathologists (Zhi‐Guang Huang and Gang Chen) who were blind to the data. The intensity of LPCAT1 for an individual sample was represented with a score that was determined by the proportion of stained cells (0 score: <5%; 1 score: 5%–25%; 2 score: 25%–50%; 3 score: 50%–75%; 4 score: >75%) and the staining degree of LPCAT1‐positive cells (0 score: no staining; 1 score: light yellow or yellow; 2 score: brown; 3 score: dark brown).^18^ An optical microscope was applied to capture the images.

#### 2.1.2 Analysis of IHC data

Violin plots of LPCAT1 expression in the IHC test were made to assist researchers in exploring whether LPCAT1 expression remained the same in cancerous and non‐cancerous tissues. An independent samples *t*‐test was used to investigate differential expression. In addition, receiver operating characteristic (ROC) curves were drawn to show the classification power of the LPCAT1 protein level in GC. An area under the curve (AUC) of >0.7 suggested the prognostic capability of the LPCAT1 protein level to some extent. The violin plots and ROC curve were drawn with the R package *ggplot2* and SPSS 22.0. Data analysis was performed with SPSS 22.0; *p* < 0.05 indicated statistical significance.

### 2.2 Expression of LPCAT1 mRNA in GC and noncancerous tissues

#### 2.2.1 High‐throughput transcriptome microarrays and sequencing data from public databases

Public databases—The Cancer Genome Atlas (TCGA), Gene Expression Omnibus, ArrayExpress database, and Sequence Read Archive—were searched for sequencing data and gene microarrays of LPCAT1 to investigate the differential expression of LPCAT1 mRNA in cancerous and normal tissues in GC. In accordance with the search strategy, inclusion and exclusion standards and the expression data of LPCAT1 were reported in our study. The terms used in the search strategy were (“malignant” OR “cancer” OR “tumor” OR “tumour” OR “neoplasm” OR “carcinoma”) AND (“STAD” OR “stomach cancer” OR “gastric cancer”). The data accord with the inclusion standard contained human biological samples, mRNA data of LPCAT1, and GC and control groups. The criteria for exclusion were as follows: (1) non‐human samples, (2) a lack of LPCAT1 expression data, and (3) no GC or control groups.

#### 2.2.2 Analysis of microarrays and sequencing data

After the public microarrays and RNA sequencing data were screened, the different datasets in the same platform were combined, and the batch effect was eliminated using the R package *sva*. We divided the data into two cohorts—GC and control groups. After screening the expression of LPCAT1 and corresponding samples in each study, *t*‐test and Wilcoxon rank sum test were used to compare the differential expression of LPCAT1 in GC and non‐tumorous tissues according to the distribution type of the expression value. The expression levels of LPCAT1 in each dataset are shown as boxplots, and the distributions of expression values are shown as ridge plots. The graphs were drawn with *ggplot2*‐derivatived packages.

To comprehensively achieve the expression tendency of LPCAT1 in GC, the sample counts, arithmetic mean of LPCAT1, and standard deviation (SD) of LPCAT1 in GC and non‐tumorous samples in the datasets were extracted and arranged in the following order: exp counts, exp mean, exp sd, ctrl counts, ctrl mean, and ctrl sd. Subsequently, the standard mean difference (SMD) and the 95% confidence interval (95% CI) were calculated using STATA 12.0. The type of effects model was determined by I‐square and chi‐square tests in the SMD model. A random‐effects model was used when high heterogeneity existed (*I* ^2^ > 50% or *p* < 0.05); if no heterogeneity was observed, a fixed‐effects model was employed. In addition, the high heterogeneity alerted us to eliminate inappropriate data by performing a sensitivity analysis. The publication bias in the SMD model was examined using Egger's test. The sensitivity analysis and publication bias of the SMD model were visualized with forest plots and funnel plots. Moreover, an SMD model with a random‐effects model was used to calculate the expression of all the protein‐coding genes in GC. Those whose mean and lower confidence intervals were greater than zero were regarded as highly expressed genes.

The ROC curves and the AUC of each study were analyzed using the R package *pROC* to evaluate the prognostic efficacy of LPCAT1 in GC. The best cutoff values of each ROC curve were calculated, and the true positive rate, false positive rate, false negative rate, and true negative rate were counted to create a summary ROC curve (SROC) with sensitivity and specificity. The SROC curve and forest plots with sensitivity and specificity were accomplished using STATA 12.0.

#### 2.2.3 Expression of LPCAT1 in GC with different clinical parameters

To detect the expression tendency of LPCAT1 in GC patients with various clinical parameters, we collected clinical data and statistics from IHC, gene microarrays, and RNA sequencing for a clinical parameter analysis, which could facilitate a profound comprehension of the clinical significance of the LPCAT1 protein and the mRNA expression level in GC. With regard to the criteria for grouping the expression status, the protein level of LPCAT1 was arranged and presented as a ranked variable. The Mann–Whitney *U* test was utilized to detect the difference in LPCAT1 protein levels by age, tissue type, gender, tumor grade, metastasis, lymphatic metastasis, tumor size, and tumor TNM stage. The sequencing and gene chip data reflected the mRNA expression level of LPCAT1. Four clinical parameters, including age (<60 years vs. >60 years), gender (male vs. female), TNM stage (Stage I–II vs. Stage III–IV), and tumor grade (highly differentiated vs. lowly differentiated) were collected from the enrolled studies. The expression of LPCAT1 was also filtered from public datasets and arranged as exp n, exp mean, exp SD, ctrl n, ctrl mean, and ctrl SD for the four clinical parameters. The clinical parameters were analyzed using an SMD model. In the same way, funnel plots were utilized to detect public bias.

#### 2.2.4 Prognostic analysis of LPCAT1 in GC

Mining TCGA and various high‐throughput datasets, we acquired large volumes of data concerning the prognosis of LPCAT1 in patients with GC. The patients with a higher expression of LPCAT1 were grouped into the high‐risk group, and the rest were defined as the low‐risk group evenly. The Cox proportional hazards model was used to calculate the hazard risk between the high‐risk group and the low‐risk group, and the log‐rank test was used to test the prognostic value of LPCAT1. HR >1 and log‐rank at *p* < 0.05 illustrated that LPCAT1 was a risk factor in patients with GC. All the tests and Kaplan–Meier (K–M) curves were carried out using the R packages *survival* and *survminer*.

### 2.3 Potential molecular mechanisms of LPCAT1 in GC

#### 2.3.1 Potential pathway of LPCAT1 in GC

Biological pathways play an important role in all kinds of tumors, such as GC. To determine which pathways LPCAT1 is involved, we estimated the enrichment scores of pathways that contained LPCAT1 and calculated the correlation between LPCAT1 and these pathways. The pathway items and genes involved based on the REACTOME subset were downloaded from the C2 sets of Molecular Signatures Database v7.5.1. The enrichment scores of the pathways in the TCGA RNA‐seq dataset and the merged GPL570 dataset were assessed using the single‐sample gene set enrichment analysis (ssGSEA) algorithm. The differently expressed pathways in the TCGA and GPL570 datasets were evaluated by the limma voom and limma algorithm, and the relevance between LPCAT1 and its located pathways was calculated by Pearson's correlation analysis. The potential tumor‐promoting pathways that LPCAT1 is involved in can be defined only if the pathway is highly expressed in GC and positively correlated to LPCAT1. The expression of pathways in GC and non‐tumorous tissue is shown by heat maps and volcano plots using R packages.

#### 2.3.2 Relation of immune microenvironment and LPCAT1 in GC

An imbalance of the proportion of immune cells in the immune microenvironment often promotes the occurrence and development of malignant tumors. The TIMER 2.0 database was used to explore the relationship between LPCAT1 and the types of immune cells. It uses the CIBERSORT algorithm to estimate the expression of 22 immune cells in the TCGA data and analyzes the correlation between LPCAT1 and immune cells.

#### 2.3.3 Regulatory network of LPCAT1 in GC

##### Selection of co‐expressed LPCAT1 genes.

To clarify the regulatory relationship of LPCAT1 in GC, one must explore the interactions between genes. Therefore, we decided to select the co‐expressed genes of LPCAT1, hoping to gain insight into the potential molecular mechanisms of LPCAT1 in GC. First, we selected tumorous samples from the TCGA data and used Pearson's correlation analysis to calculate the correlation between LPCAT1 and genes. *R* > 0 and *p* < 0.05 suggest significant co‐expressed genes. Then, highly expressed genes in GC samples based on the SMD model were intersected with the co‐expressed genes of LPCAT1. To ulteriorly expound on the regulatory relationship of LPCAT1 in GC, we used the genes in the same pathway as in LPCAT1.

##### The regulatory relationship of co‐expressed genes and LPCAT1

Cluster analysis based on the t‐distributed stochastic neighbor embedding (t‐SNE) algorithm was applied to detect the discriminatory power of co‐expressed genes between GC and nontumorous tissue. A heat map was constructed to intuitively display the expression of the co‐expressed genes of LPCAT1 in GC. The two analyses were both based on TCGA data. To investigate the regulatory network of LPCAT1‐related genes, we evaluated the protein–protein interaction (PPI) network of co‐expressed genes using the STRING (https://cn.string‐db.org/) online database. The top 10 closely linked genes were calculated using the cytohubba algorithm.

To further analyze the regulation patterns and investigate the regulation mechanism of carcinogenesis of LPCAT1 in GC, we prognosed the transcription factors (TFs) of LPCAT1 in GC. The gene regulatory network (GRN) database used the SCENIC pipeline to identify the TF–target pairs based on 72 different single‐cell conditions and 71 bulk conditions, including GC tissue. The potential TFs of LPCAT1 in GC were prognosed from GRNdb and were intersected with highly expressed genes of GC and co‐expressed genes of LPCAT1. To validate the regulatory pattern of TFs and LPCAT1, public ChIP‐seq data with the TFs marked were downloaded from the Cistrome database and visualized.

### 2.4 In vitro experiments of LPCAT1 in a GC cell line

#### 2.4.1 Cell culture and treatment

The human AGS cell line was acquired from the Institute of Biochemistry and Cell Biology of the Chinese Academy of Sciences. The AGS cells were cultured in DMEM/F‐12 medium (Gibco) with 10% fetal bovine serum (Gibco), 100 U/mL penicillin (Gibco), and 100 μg/mL streptomycin (Gibco). RNA isolation and RT‐qPCR assays were carried out to detect the silence efficiency of LPCAT1‐siRNA in AGS cells. LPCAT1‐siRNA was obtained from RIBOBIO, and the primer sequences of LPCAT1 were as follows: forward, CATGAGGCTGCGGGGATG; reverse, TCATGAGGGCCACCTGGG.

A total of 0.5 mL of 0.25% trypsase was added into the AGS cells of the logarithmic growth phase. After the cells detached, the whole culture solution was added into the cells, and the cell suspension was centrifugated for 3 min. We then adjusted the cell density to 2.5 × 10^5^/mL and added it into a 6‐well plate. The cells in the 6‐well plate were generated in CO_2_ culture at 37°C. After 1 day, the cell count in each plate was 6 × 10^5^. The AGS cells were divided into the blank control (CK) group, the negative control (NC) group, and the transfection group. Real‐time fluorescent quantitative polymerase chain reaction (RT‐qPCR) was performed, and the 2^−ΔΔCt^ value was utilized to detect the silence ability of siRNA. The GAPDH gene was selected as the internal reference gene.

#### 2.4.2 Migration assay and invasion assay

The AGS cells were divided into the control check (CK) group, NC group, and transfection groups and used to conduct the migration and invasion assays. The AGS cells in the logarithmic growth phase were separated by adding 0.5 mL of 0.25% trypsase and then centrifuged for 5 min. After centrifugation, we added serum‐free medium into the AGS cells and adjusted the cell density to 3 × 10^5^/mL. The upper chambers in a transwell chamber were filled with 100 μL AGS cells, and the lower chambers were added with complete medium. For the invasion assay, the Transwell chambers were enveloped using Matrigel basement membrane gel. The Transwell chambers were placed in a 37°C CO_2_ incubator. After incubation for 48 h, the cells were removed and fixed using 4% paraformaldehyde. The transfection group AGS cells in each well were added 2.2 pmol LPCAT1‐siRNA. Finally, we randomly selected five visual fields and used them to exhibit. To qualitatively estimate the degree of cell invasion and migration, we read the optical density (OD) values at 590 nm for each chamber using a microplate reader.

### 2.5 Statistical analysis

The statistical analysis in our study were performed by SPSS 22.0, R software, STATA 12.0 and GraphPad Prism 9.0. For the statistical description, ratio was used to describe classification variable, and mean ± SD OR median ± quartile was used to describe continuous variable. Chi‐square test and Fisher exact test were used to compare the difference of classification variable, and *t*‐test and Wilcoxon rank sum test were used to compare the difference of continuous variable. Two‐sided *p* < 0.05 indicated significant statistical difference.

## 3 RESULTS

### 3.1 Expression of LPCAT1 protein in GC

**FIGURE 1:**
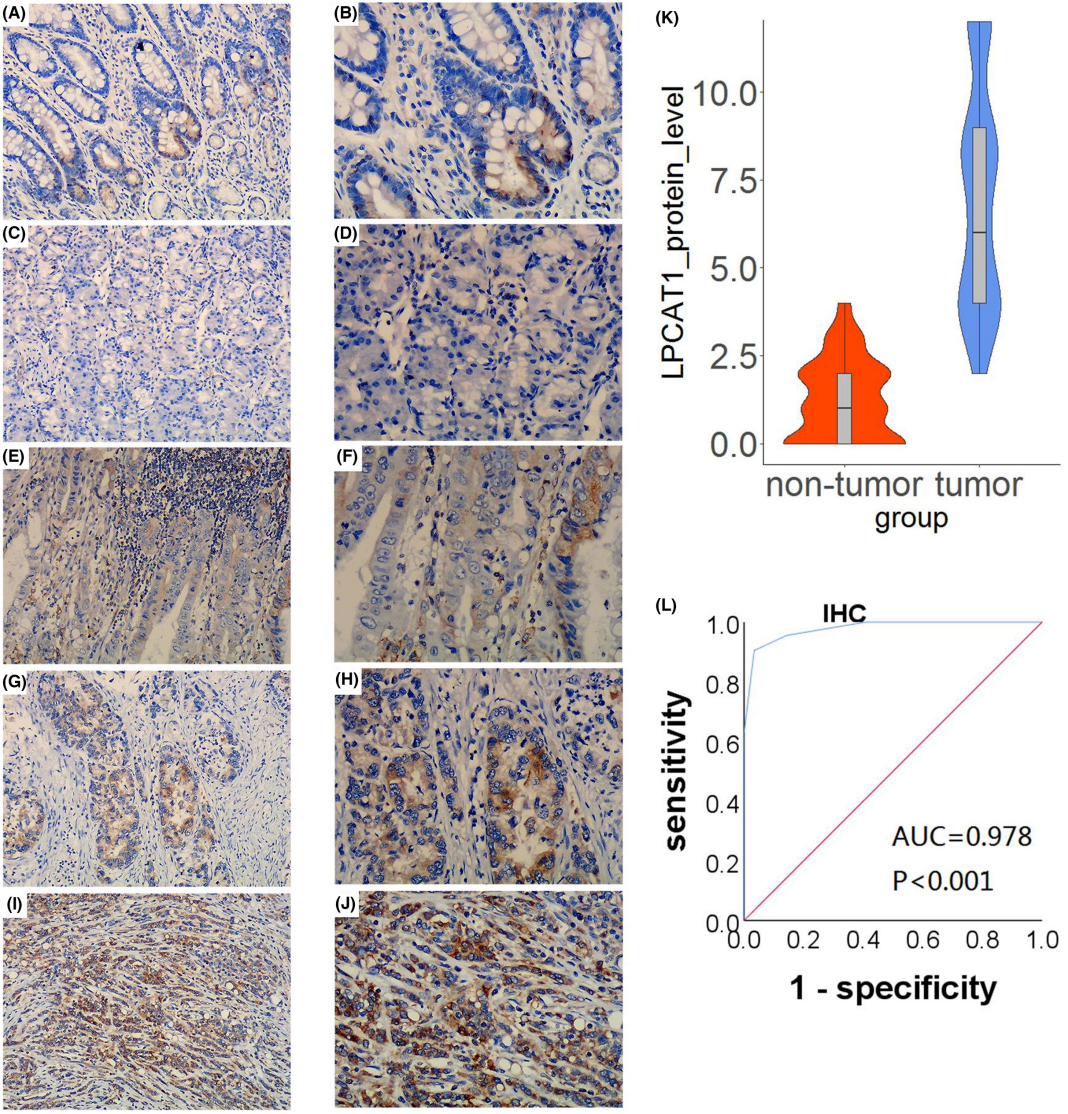
High protein level of LPCAT1 in GC compared to non‐tumorous stomach tissue. (A,B) LPCAT1 staining in a part of glandular cells in chronic gastrtis tissues (×100, ×200). (C,D) LPCAT1 staining in normal stomach tissue (×100, ×200). (E,F) LPCAT1 staining in grade I stomach cancer tissue (×100, ×200). (G,H,I,J) LPCAT1 staining in grade II stomach cancer tissue (×100, ×200, ×100, ×200). (K) The quantitative analysis showed highly‐expressed protein level of LPCAT1 in GC. GC tissue was reflected in blue violin chart, red was non‐tumor tissue. (L) AUC of ROC curve was 0.978, and *p* value.

To explore the protein level of LPCAT1 in GC, a total of 326 cases were involved in the IHC test, with 179 GC cases and 147 non‐cancerous cases. As shown in Figure 1, the protein level of LPCAT1 in GC was higher than in non‐tumorous tissue. LPCAT1 showed almost no staining (only a small portion was stained brownish yellow) in the gastritis tissues and normal gastric tissue (Figure 1A–D), whereas LPCAT1 was obviously stained brown in the GC tissues (Figure 1E–J). Compared with the GC Grade I tissue (Figure 1E,F), LPCAT1 in the Grade II tissues was extensively stained dark brown (Figure 1G–J). After the quantitative test, LPCAT1 showed a higher expression in GC tissues than in non‐cancerous ones (*p* < 0.001), which is shown in the violin plots (Figure 1K). The ROC curves revealed that AUC = 0.978 and *p* < 0.001, indicating that LPCAT1 could effectively predict the course of GC (Figure 1L).

### 3.2 Expression of LPCAT1 mRNA in GC

#### 3.2.1 Analysis of RNA‐seq and gene microarrays

From the public database, the studies regarding the expression of LPCAT1 were enrolled. According to the inclusion standard, a total of 35 studies from 15 high‐throughput platforms were included in our research (Table S1). After merging the expression matrix and removing the batch effect, these contributed to 2667 cases of samples, of which 1727 were GC cases and 940 were non‐cancerous cases, which were sufficient for the researchers to investigate the expression of LPCAT1 in GC.

#### 3.2.2 High expression of LPCAT1 mRNA in GC

**FIGURE 2:**
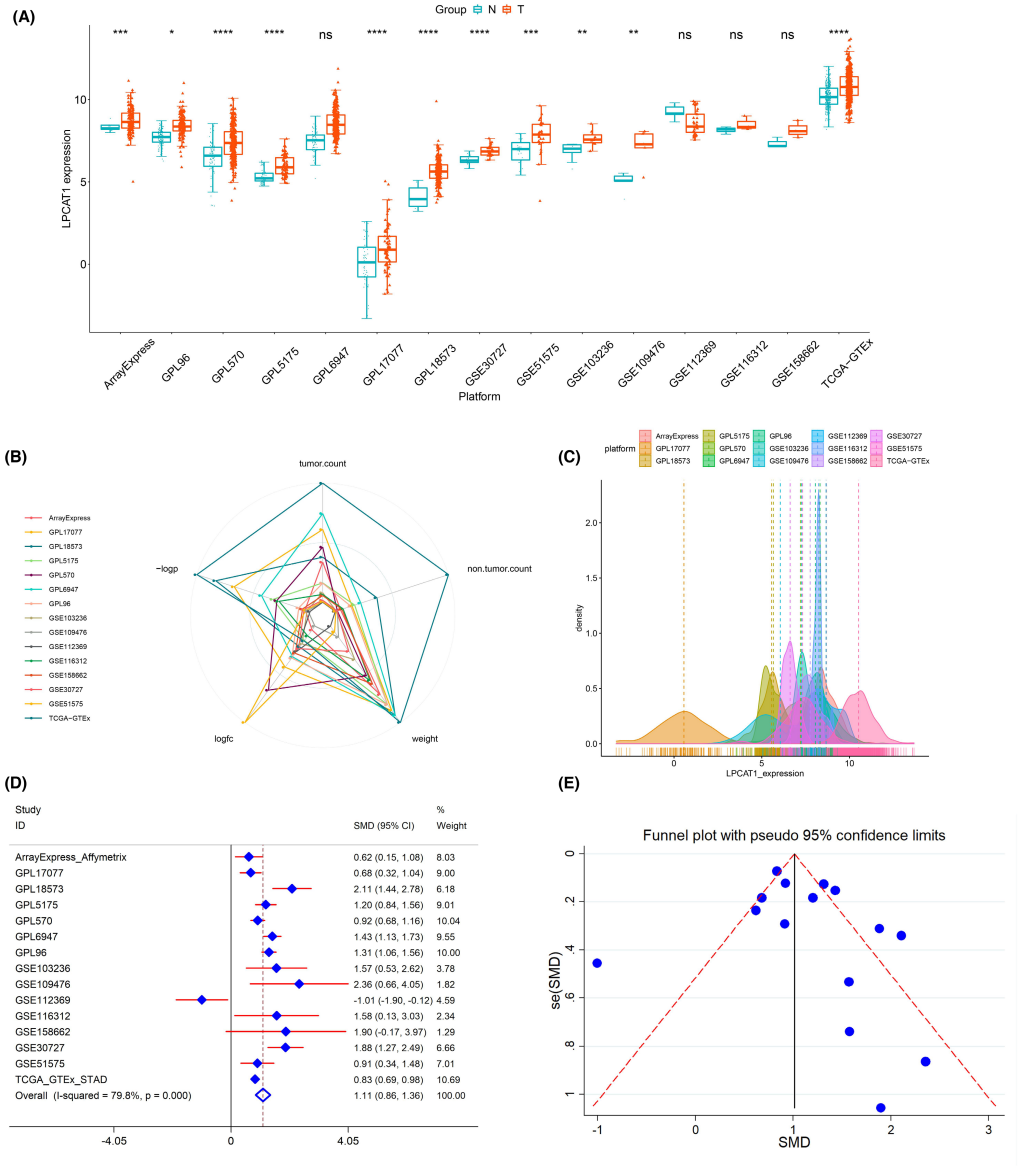
The expression of LPCAT1 in GC. (A) The expression of LPCAT1 in each study was performed with boxplots. LPCAT1 was highly expressed in most of the studies except GPL6947, GSE109476, GSE112369 and GSE158662. (B) The expression information including SMD model weight, logFC, adjust p value, tumor counts and non‐tumor counts of each study was exhibited by radar chart. (C) The expression values of LPCAT1 in the all datasets conformed to the Gaussian distribution. (D,E) SMD with random effect model showed LPCAT1 was highly expressed in GC tissue compared to non‐tumorous tissue. No public.

The expression of LPCAT1 in GC and noncancerous tissues for each dataset was visualized with box plots (Figure S1A–O), which would improve our understanding of its role in GC. According to the variance analysis, LPCAT1 was highly expressed in GC tissue compared with nontumorous tissue in most of the datasets (Figure 2A). The information on LPCAT1 expression in each dataset was reflected in the radar chart. The TCGA dataset clearly had the largest number of tumor and nontumorous samples (Figure 2B). The expression values of LPCAT1 in most of datasets conformed to Gaussian distribution (Figure 2C) (Table S2). Integrated analysis showed that LPCAT1 was highly expressed in GC tissue compared with non‐tumorous tissue with a SMD = 1.11 and 95% CI = 0.86–1.36 (Figure 2D). The random effect model was used to weaken the heterogeneity caused by some datasets (*I* ^2^ = 79.8%, *p* < 0.0001). No public bias was observed in the SMD model with *p* = 0.342 by Egger's test (Figure 2E) (Table S3). In addition, 5193 highly expressed genes of GC were calculated using the SMD algorithm (Table S4).

The ROC curves for each study were constructed using SPSS 22.0, and the AUC and *p* values were used to help evaluate the discriminatory power of LPCAT1 in GC and non‐tumorous tissue (Figure S2A–O). Most of the ROC curves showed statistical significance, with *p* < 0.05. The SROC curve indicated that highly expressed LPCAT1 could be used as an effective way to predict the medical outcomes of GC (AUC = 0.85, 95% CI = 0.8–0.88). Moreover, the high specificity of the SROC model (specificity = 0.81, 95% CI = 0.73–0.88) showed a high accuracy of LPCAT1 (Figure S3).

#### 3.2.3 The clinical value of LPCAT1 in GC

##### The relation of LPCAT1 and clinical parameters

**FIGURE 3:**
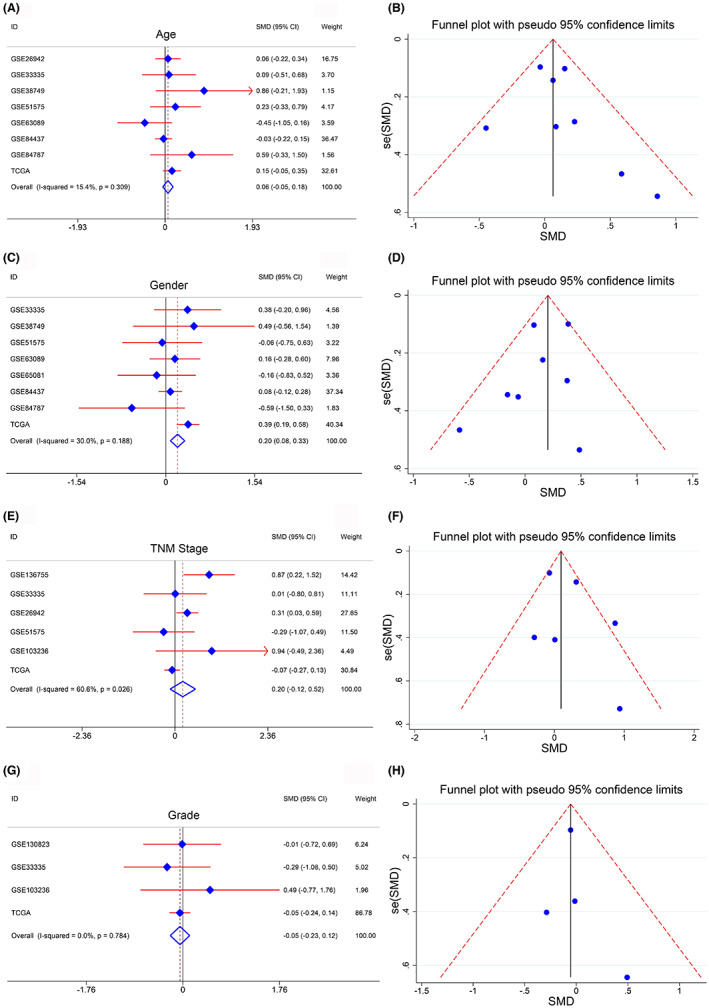
The clinical parameter analysis of LPCAT1. (A–H) Four clinical parameters (age, gender, TBN stage and tumor grade) were enrolled and the expression of LPCAT1 in each parameter were calculated with SMD model. Higher expression of LPCAT1 was only observed in the group of male, as compared to female. No difference of LPCAT1 expression was noted when considering other clinical parameters.

The clinical significance of LPCAT1 could be analyzed owing to the sufficient clinical parameters provided by the in‐house data and public cohorts. The results for the in‐house cohort revealed that the protein level of LPCAT1 was solely connected with the histological type (Table S5). Furthermore, LPCAT1 was only highly expressed in male patients with GC based on integrated analyses, with SMD = 0.20 (95% CI = 0.08–0.33; Figure 3C,D). Neither of the integrated analyses of LPCAT1 in age (SMD = 0.06, 95% CI = −0.05–0.18), TNM stage (SMD = 0.20, 95% CI = −0.12–0.52), or tumor grade (SMD = −0.06, 95% CI = −0.23–0.12) had a significant difference (Figure 3A,B, E–H). No public bias was observed in the four SMD models. In summary, LPCAT1 could be stably expressed in GC in various situations.

##### Prognostic analysis of LPCAT1 in GC

The TCGA cohort, GSE38749, GSE84426, and GSE84433, which included prognosis information, overall survival time, expression of LPCAT1, and status of patients with GC, were extracted to carry out Cox regression and log‐rank tests. However, the four K–M plots showed that LPCAT1 was not correlated to the survival time of patients with GC, with a log‐rank of *p* < 0.05 (Figure S5).

### 3.3 Investigation into the molecular mechanism of LPCAT1

#### 3.3.1 The potential pathways of LPCAT1 in GC

**FIGURE 4:**
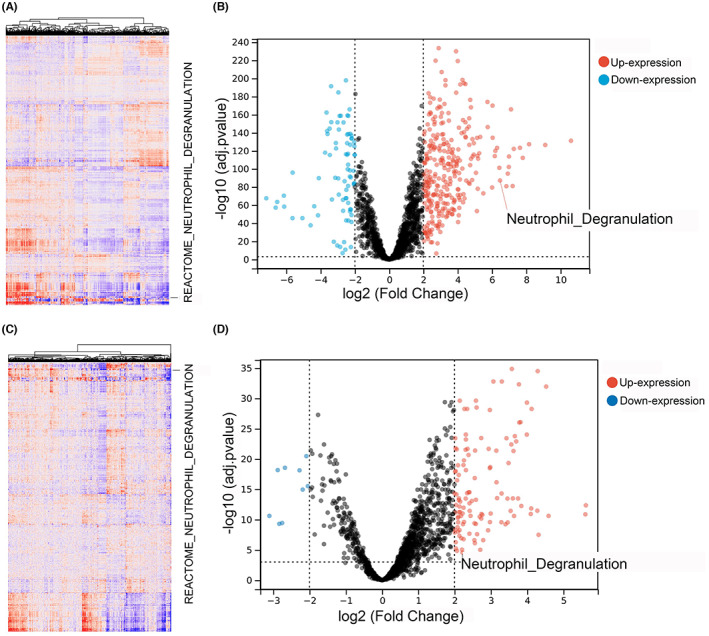
Pathway analysis of LPCAT1 in GC. (A,B) As a LPCAT1‐related pathway, neutrophil degranulation pathway was highly expressed in GC based on TCGA dataset. (C,D) And the analysis based on GPL570 dataset corroborate the high expression level of neutrophil degranulation pathway.

From the REACTOME pathway subset, nine pathways involving LPCAT1 were taken advantage of (Table S6). The enrichment scores of all REACTOME pathways were assessed based on the TCGA and GPL570 datasets and were visualized with heat maps, and the differently expressed pathways were calculated (Figure 4A–D). Only the neutrophil degranulation pathway was both highly expressed in GC (logFC = 6.47, adj *p* < 0.0001) and positively related to LPCAT1 (*r* = 0.30, *p* < 0.0001). The neutrophil degranulation pathway was exhibited in heat maps and volcano plots, and the genes in the pathway were selected for subsequent analysis.

#### 3.3.2 Immune microenvironment analysis of LPCAT1 in GC

**FIGURE 5:**
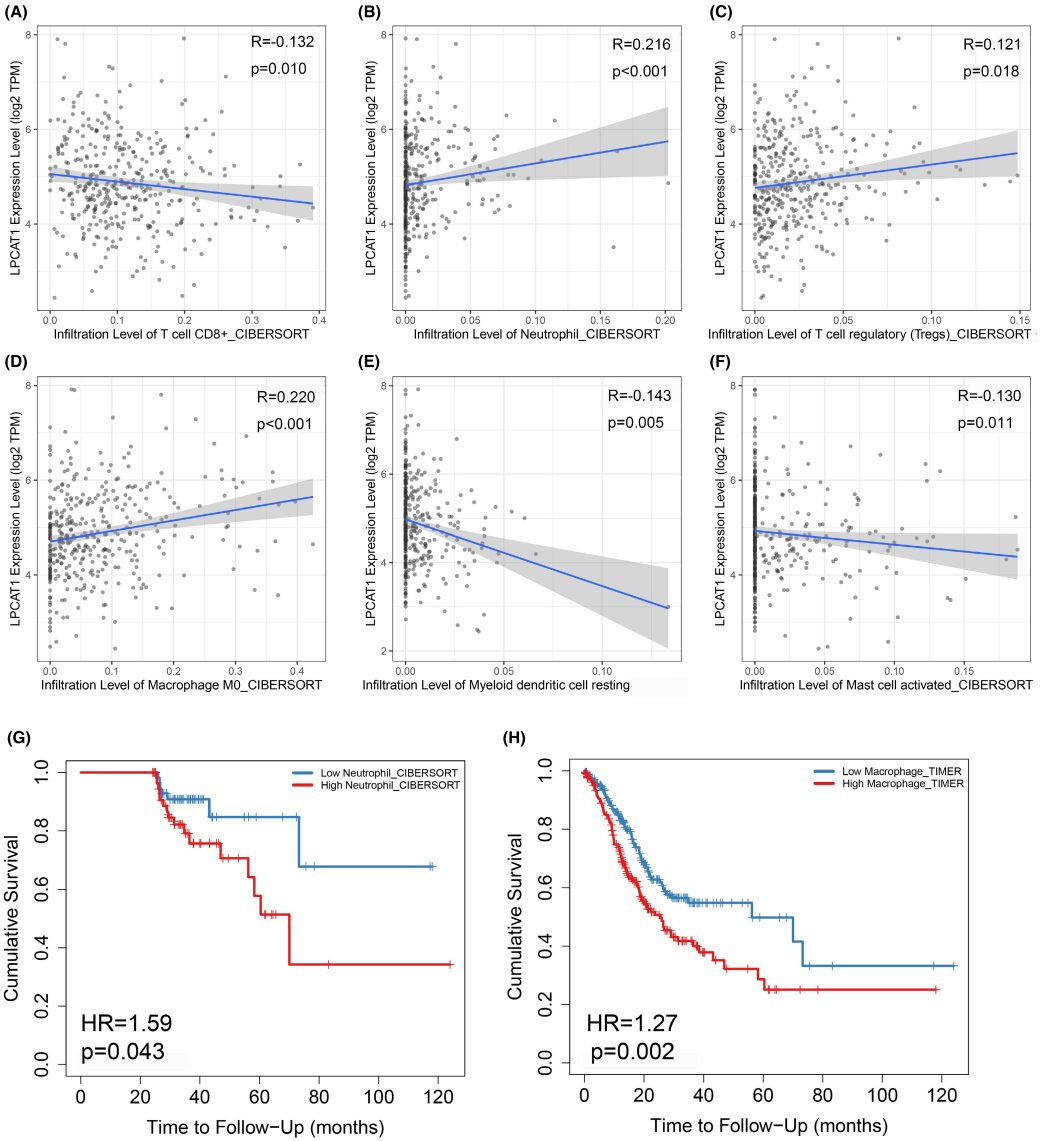
LPCAT1 correlated to immune microenvironments in GC. (A–F) Total six immune cells were interrelated to LPCAT1 in GC. The X‐axis was the expression of immune cells and the Y‐axis was the expression value of LPCAT1. The blue fit line with 95% CI was performed and reflected the relation of LPCAT1 and immune cells. (G,H) COX regression analysis showed Neutrophil and macrophage were the risk factor of GC patient.

In the immune microenvironment, six immune cells were interrelated with LPCAT1 in GC. CD8^+^ T cells, myeloid dendritic cells, and mast cells were markedly negatively correlated to LPCAT1 (*r* < 0, *p* < 0.05); by contrast, neutrophils, regulatory T cells, and macrophages were markedly positively correlated to LPCAT1 (*r* > 0, *p* < 0.05) (Figure 5A–F). Moreover, as positively related immune cells of LPCAT1, neutrophils and macrophages were risk factors of GC, where their high expression indicates a poor outcome in patients with GC (Figure 5G,H).

#### 3.3.3 The regulatory mechanism of LPCAT1 in GC

**FIGURE 6:**
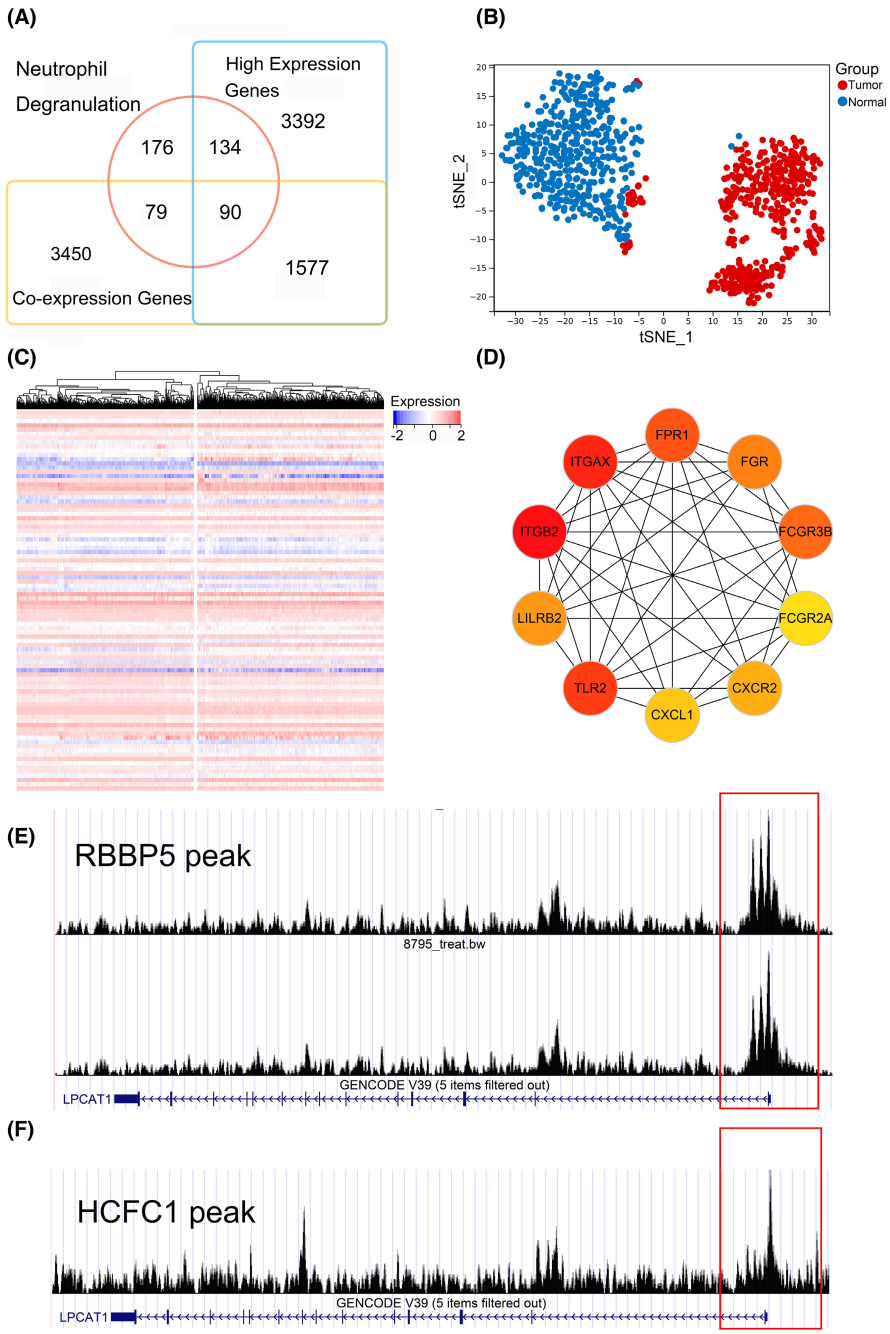
The analysis of co‐expressed genes and regulatory mechanism of LPCAT1 in GC. (A) The LPCAT1‐related genes were overlapped from neutrophil degranulation pathway, co‐expressed genes of LPCAT1 and up‐regulated genes of GC. (B,C) Clustering diagram and heatmap showed the co‐expressed genes of LPCAT1 could commendably discriminate the tumor and non‐tumorous samples. (D): The PPI network indicated the top 10 genes most related to LPCAT1. (E,F) RBBP5 and HCFC1 could accelerate the expression of LPCAT1 and the peaks were marked in the frame.

A total of 5197 co‐expressed genes were filtered from the TCGA dataset via Pearson's correlation analysis. To acquire more accurate regulatory information of LPCAT1 and the co‐expressed genes, the over‐expressed genes of GC in the neutrophil degranulation pathway were overlapped with the co‐expressed genes of LPCAT1. Finally, a total of 91 genes were filtered and seemed to be the LPCAT1‐related gene group (Figure 6A).

The co‐expressed genes of LPCAT1 could commendably discriminate the tumor and non‐tumorous samples based on the t‐SNE cluster and consensus cluster (Figure 6B,C). The PPI network was analyzed based on the LPCAT1‐related gene group, and the top 10 closely linked genes were calculated (Figure 6D). For the upstream regulatory mechanisms, RBBP5 and HCFC1 were the potential TFs targeted to LPCAT1, and the regulatory relationship was validated by ChIP‐seq peaks (Figure 6E,F).

### 3.4 The tumor‐promoting effect of LPCAT1 verified by in vitro experiment

**FIGURE 7:**
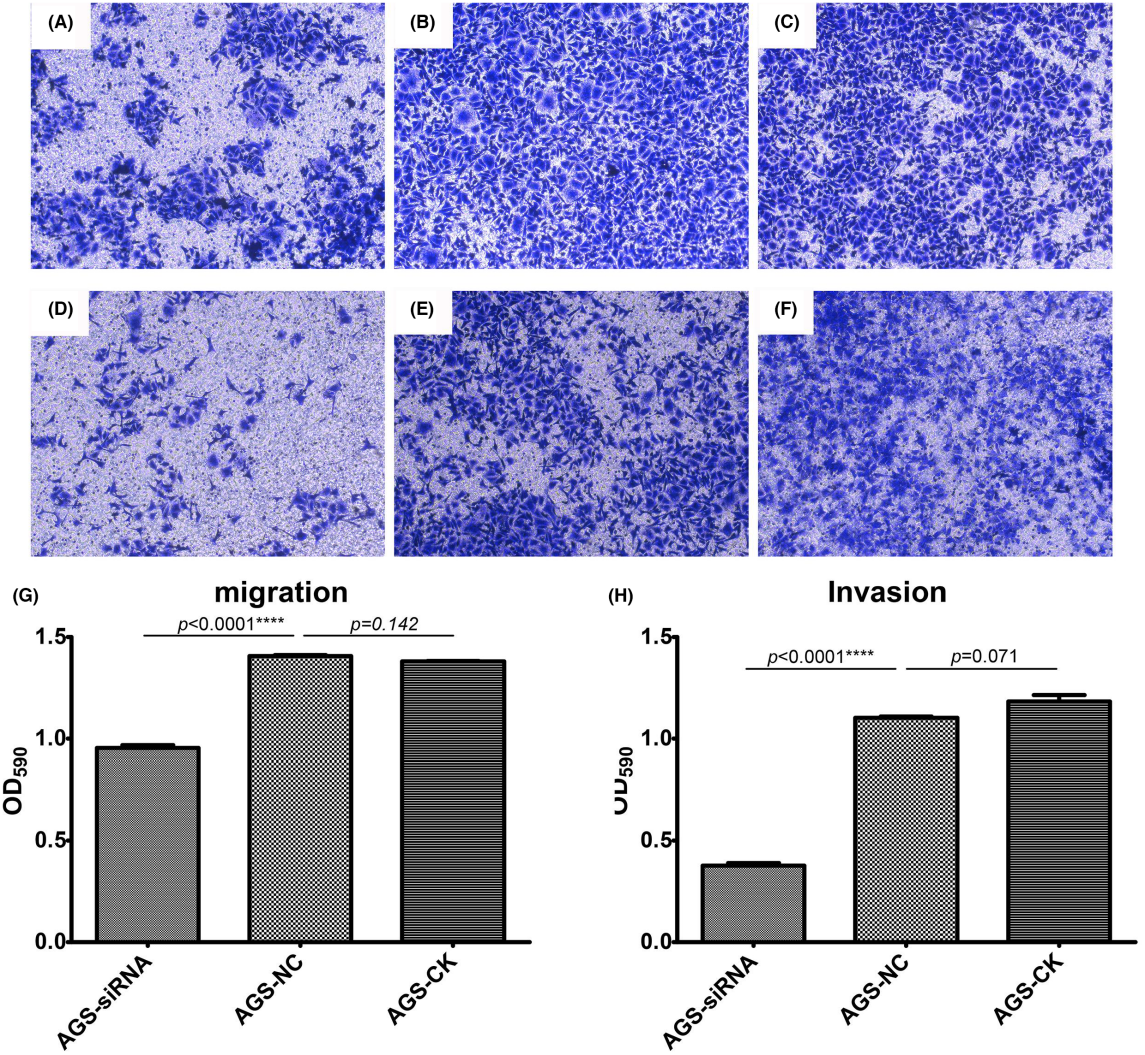
In vitro experiment of LPCAT1 in AGS cells. (A–C) The typical pictures in the migration assay were used to exhibited. Picture A–C were the siRNA group, NC group and CK group. (D–F) The siRNA group, NC group and CK group of the invasion assay. (G,H) The OD590 value of siRNA group was significantly lower than the NC group and CK group in the migration assay and the invasion assay.

In light of the 2^−ΔΔCt^ value of RT‐qPCR, the expression of LPCAT1 with siRNA transfection was significantly lower than that in the CK and NC groups (Figure S5). This demonstrated that the antagonistic effect of siRNA was sufficiently strong. Figure S6A–D show the amplification curves and the dissolution curves of GAPDH and LPCAT1. The migration and invasion rates of AGS cells in the siRNA‐transfected group were markedly lower than those of the CK and NC groups. The fields in the migration and invasion assays of each group were randomly selected and displayed (Figure 7A–F). The rates of migration and invasion in each group were also quantitatively analyzed (Figure 7G,H).

## 4 DISCUSSION

LPCAT1, is mainly located on the cell membrane and participates in biological functions, such as lipid metabolism and membrane lipid remodeling.^21^ LPCAT1 in rats is mostly situated on the surface of pulmonary alveoli, which is generated by Type II cells; it maintains the stability of alveolar lipids and ensures normal respiration.^22^, ^23^ In one study, LPCAT1 was expressed in human lungs at an obviously high level, promoting the formation of alveolar lipids and the regulation of respiratory functions.^24^ Moreover, some authors have pointed out that LPCAT1 in rats is involved in the regulation of photoreceptors in the retina.^25^, ^26^ In recent years, reports on the relationship between LPCAT1 and cancers have increased; nonetheless, few reports have focused on LPCAT1 in GC. A study published in 2016 found LPCAT1 is highly expressed in gastric adenocarcinoma; however, the data were from a single source, and the study concentrated solely on the protein level of LPCAT1 without analyses of its mRNA and its molecular mechanism in GC.^20^ As a result, in the current study, the function of LPCAT1 in GC was set forth across the board. The high mRNA and protein expression levels of LPCAT1 in GC were authenticated by a large sample size of public high‐throughput data and in‐house immunohistochemical staining. A large number of computational biology analyses have shown that LPCAT1 could accelerate the process of GC via the active neutrophil degranulation pathway and a dysfunctional immune microenvironment. The in vitro experiments determined that LPCAT1 could boost the invasion and migration of GC cells. In addition, the regulatory mechanism of LPCAT1 was also clarified and corroborated via a biological algorithm and ChIP‐seq data.

First, we tested the protein levels of LPCAT1 in different gastric tissues using IHC, and the results demonstrated that the protein levels of LPCAT1 were remarkably higher in GC than in noncancerous tissues, which was consistent with previous studies. The study in 2016 showed LPCAT1 was highly‐expressed in GC and might boost the GC by regulating lipid metabolism, and our result confirmed the expression status and cancer promoting effect of LPCAT1 in GC.^20^ Furthermore, ROC curves were generated to analyze the classified ability of LPCAT1 in GC, which has not been performed in prior studies. In many clinical diagnostic tests, ROC curve is used to check the diagnostic performance of potential markers and a more AUC value representing a higher diagnostic capability.^27^ The result showed that LPCAT1 could effectively forecast the outcomes of GC (AUC = 0.978, *p* < 0.001). As we know, all the genes need translate to protein to carry out its function. And highly‐expressed status of LPCAT1 had been reported in various cancers. So we think that LPCAT1 may play an important role in GC.

Then the highly‐expressed value of LPCAT1 in GC was verified. The mRNA expression level of LPCAT1 are our sections of interest because the high‐throughput RNA microarrays and RNA sequence are increasingly popular in recent years. And all the computational biology methods need mRNA expression data.^28^, ^29^ Hence, a large number of public high‐throughput GC data were retrieved and utilized. The SMD model was used to calculate the differently expressed status based on the mRNA arrays and mRNA sequence data because in traditional difference analyses, such as multivariate ANOVA or limma, may leave out some critical genes according to the rigorous *p* value.^30^, ^31^ Hence, the expression of LPCAT1 based on SMD model is better than the traditional algorithms. Surprisingly, the mRNA values of LPCAT1 in GC tissue are higher than in nontumorous tissue and consistent with the IHC results. On most occasions, mRNA level and protein level in cells always show a positive correlation trend, so it can be ulteriorly determined that LPCAT1 is highly‐expressed in GC.^32^ Therefore, we can boldly speculate that LPCAT1 plays a role in promoting cancer in GC.

Due to the high expression state of LPCAT1 in GC, we attempted to analyze the clinical value of LPCAT1 in patients with GC based on protein and mRNA levels. No difference in the protein level of LPCAT1 was observed in the clinical parameters included in the in‐house IHC data. It illustrates that we cannot use LPCAT1 to make some clinical judgments. Then the relations of LPCAT1 mRNA and clinical parameters in public cohorts were also calculated by SMD model. LPCAT1 is highly‐expressed in female patients compared to male patients. Some researches showed gender‐specific genes had different expression status in male or female patients and exert different cancer promoting effects.^33^, ^34^, ^35^ So LPCAT1 may conduct its cancer‐promoting function in female patients more than in male patients. However, LPCAT1 was not differently expressed in the groups according to age, TNM stage, and tumor grade. It suggests that LPCAT1 can balance expression to continuously boost the tumor in patients with GC in various states.

Computational biology methods were utilized to analyze by which means LPCAT1 promotes the progression of GC. Pathway analysis with ssGSEA algorithm can assist us in better understanding the mechanism of LPCAT1 in GC.^36^ In our study, up to nine studies with 310 GC samples and 91 nontumorous samples belonged to the GPL570 platform. To make the result more convincing, we used the TCGA cohort and merged the GPL570 dataset used to analyze the expression of pathways. Two pathways (innate immune system pathway and neutrophil degranulation pathway) were highly expressed in the GC samples and positively correlated with LPCAT1 and the neutrophil degranulation pathway eventually seemed to be the pathway related to LPCAT1 because it is contained in the innate immune system pathway. Neutrophil degranulation pathway is an important biological pathway for maintaining the homeostasis of the immune system. Some tumor cells, including GC cells, can cause the immune system disturbances such as T lymphocytes, B lymphocytes and neutrophil.^37^, ^38^ A research indicated that neutrophil degranulation might exert immunosuppressive role in lung adenocarcinoma.^39^ And lots of oncogenes were reported to disturb the immune‐related pathways including the neutrophil degranulation pathway in GC.^40^ So LPCAT1 may positively affect the immune circumstances to perform its tumor‐promoting role in GC. Interestingly, the immune microenvironment analysis suggests that LPCAT1 can increase neutrophils, regulatory T cells, and macrophages and decrease CD8^+^ T cells, myeloid dendritic cells, and mast cells. Neutrophils, regulatory T cells and CD8^+^ T cells, which are typical immunosuppressive cells in various cancers.^41^ In conclusion, LPCAT1 plays a negative role in GC by boosting the neutrophil degranulation pathway and disturbing the immune microenvironment.

To improve the biologic mechanisms of LPCAT1 in GC, the regulatory mechanisms of LPCAT1 in GC are also explored using computational biology methods and multi‐omics data. Co‐expressed genes are strong‐related genes and always affect a synergistic effect in malignancies.^42^, ^43^ In accordance with abundant previous studies, the co‐expressed genes in our study were calculated using correlation analysis based on high‐throughput data.^43^, ^44^ LPCAT1‐related genes can perfectly separate the GC and non‐tumorous tissue so we consider that LPCAT1 can combine these genes to play a role in promoting cancer. For the upstream regulation mechanism, the potential transcription factors, RBBP5 and HCFC1, are considered to boost the expression of LPCAT1. As a part of epigenetic regulation, transcription factors play an important role in the occurrence and development of malignant tumors, they can boost or reduce the expression of tumor‐associated genes.^45^ RBBP5 encodes a ubiquitously expressed nuclear protein which belongs to a highly conserved subfamily of WD‐repeat proteins, and it leads a progression in multiple malignancies especially in prostate cancer.^46^, ^47^ HCFC1 is a member of the host cell factor family, and it is also active in many diseases and exerts its transcriptional regulatory role.^48^, ^49^ For the first time, we found that RBBP5 and HCFC1 can boost the expression of LPCAT1 to accelerate tumor progression in GC.

To verify whether LPCAT1 can speed up the progress of GC, we designed a series of in vitro experiments. The AGS cell line was derived from human gastric adenocarcinoma tissue and has been used in most studies.^50^ In our study, an AGS cell line was constructed for the migration and invasion assays. After transfecting the siRNA of LPCAT1, the rates of invasion and migration were retarded. In summary, LPCAT1 is highly expressed in GC, and over‐regulated LPCAT1 can boost GC progression.

However, the present study has some limitations. First, the integrated analysis of 2667 cases unavoidably entailed heterogeneity to some extent, which could be attributed to a wide range of factors, such as the test techniques for LPCAT1 expression, the area from which the samples were obtained, sex, age, tumor stage, and types of GC samples (frozen/fresh tissues, formalin‐fixed and paraffin‐embedded tissues, or blood tissues). Hence, large‐scale clinical experiments are urgently needed in the future for subgroup analyses to investigate the source of heterogeneity. Second, more in vivo and in vitro experiments are needed to prove the function of LPCAT1.

## 5 CONCLUSION

The mRNA and protein of LPCAT1 are highly expressed in GC relative to nontumorous tissue, and LPCAT1 can accelerate the invasion and migration of GC by boosting the neutrophil degranulation pathway and disturbing the immune microenvironment.

## AUTHOR CONTRIBUTIONS

**Zu‐Xuan Chen:** Data curation (lead); formal analysis (lead); methodology (lead); resources (lead); visualization (lead); writing – original draft (lead). **Liang Liang:** Data curation (equal); formal analysis (equal); investigation (equal); methodology (equal); resources (equal); software (equal). **He‐Qing Huang:** Data curation (equal); formal analysis (equal); methodology (equal); software (equal); visualization (equal). **Jian‐Di Li:** Investigation (equal); methodology (equal); software (equal). **Rong‐Quan He:** Data curation (equal); resources (equal); software (equal); validation (equal). **Zhi‐Guang Huang:** Software (equal); visualization (equal). **Rui Song:** Formal analysis (equal). **Gang Chen:** Validation (equal); writing – review and editing (equal). **Jian‐Jun Li:** Data curation (equal). **Zheng‐Wen Cai:** Conceptualization (lead); writing – review and editing (equal). **Jie‐An Huang:** Conceptualization (lead); funding acquisition (lead); project administration (lead).

## FUNDING INFORMATION

This work was supported by the Natural Science Foundation of China (grant no. 81760516), the Nature Science Foundation of Guangxi (grant no. 2019GXNSFAA185030), the self‐funded scientific research project of western medicine category of Guangxi Zhuang Autonomous Region Health Commission (grant no. Z‐A20220611).

## CONFLICT OF INTEREST STATEMENT

The authors declared no potential conflicts of interest with respect to the research, authorship, and/or publication of this article.

## Supporting information


Figure S1.
Figure S2
Figure S3
Figure S4
Figure S5
Figure S6

Table S1.

Table S2.

Table S3.

Table S4.

Table S5.

Table S6.

## Data Availability

The raw data can be obtained from the corresponding author or download from the public database.
