# Supplementary material for: LPCAT1 enhances the invasion and migration in gastric cancer: Based on computational biology methods and in vitro experiments
Source: Cancer Med. 2023 May 15;12(12):13438–54. doi: 10.1002/cam4.5991 (PMC10315734; doi:10.1002/cam4.5991)
Supplement: Supplementary file 1 — Figure S1. Figure S2 Figure S3 Figure S4 Figure S5 Figure S6 [file CAM4-12-13438-s005.pdf]

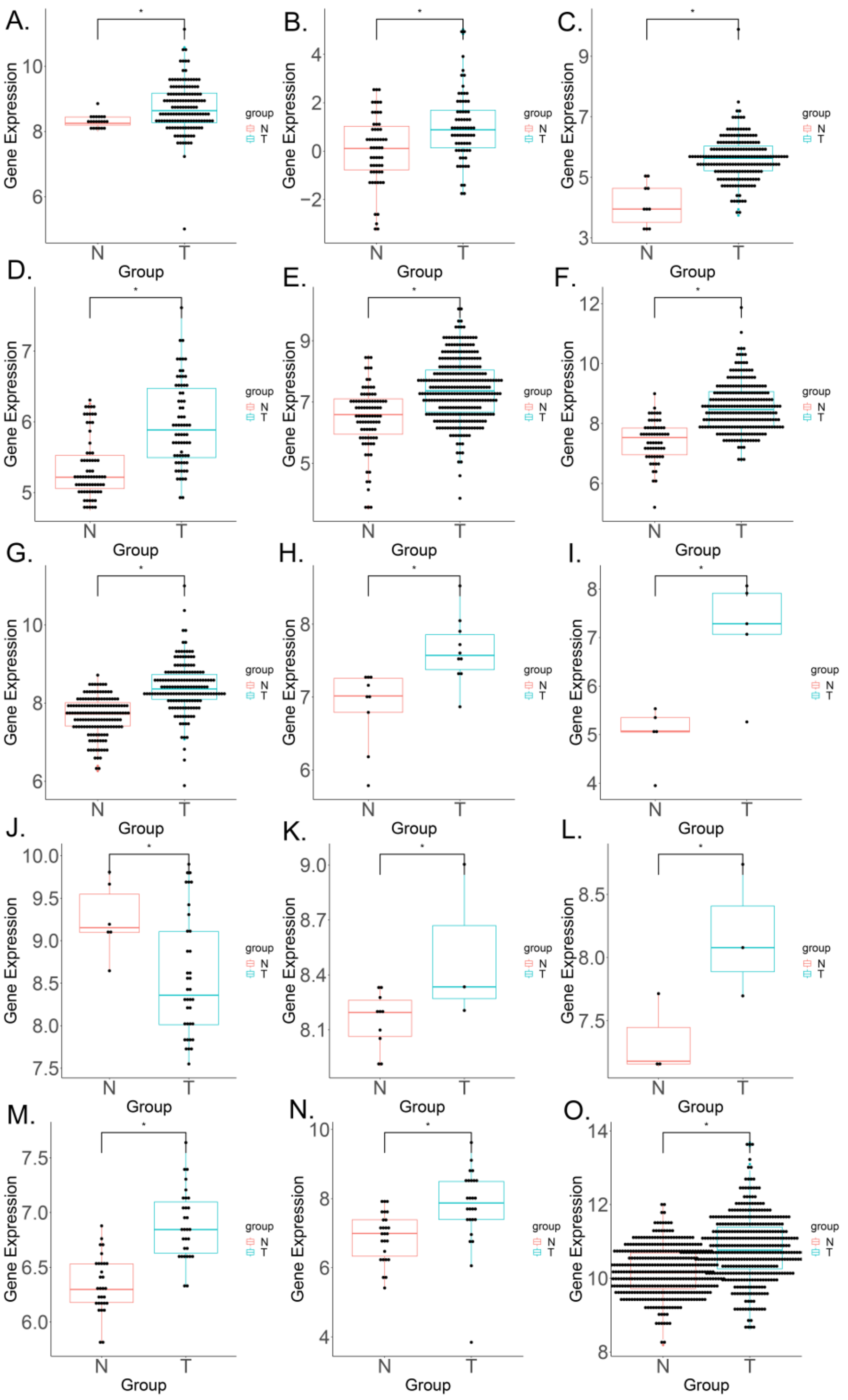

Supplementary Figure 1 Violin charts and box plots were performed for each study. The width of violin was reflected the samples enrichment degree. Box plots reflected mean value and quartile value.

(A): ArrayExpress_Affaymetrix (B): GPL17077 (C): GPL8573 (D): GPL5175 (E): GPL570 (F): GPL6947 (G): GPL96 (H): GSE103236 (I): GSE109476 (J): GSE112369 (K): GSE116312 (L): GSE158662 (M): GSE30727 (N): GSE51575 (O): TCGA_GTEx

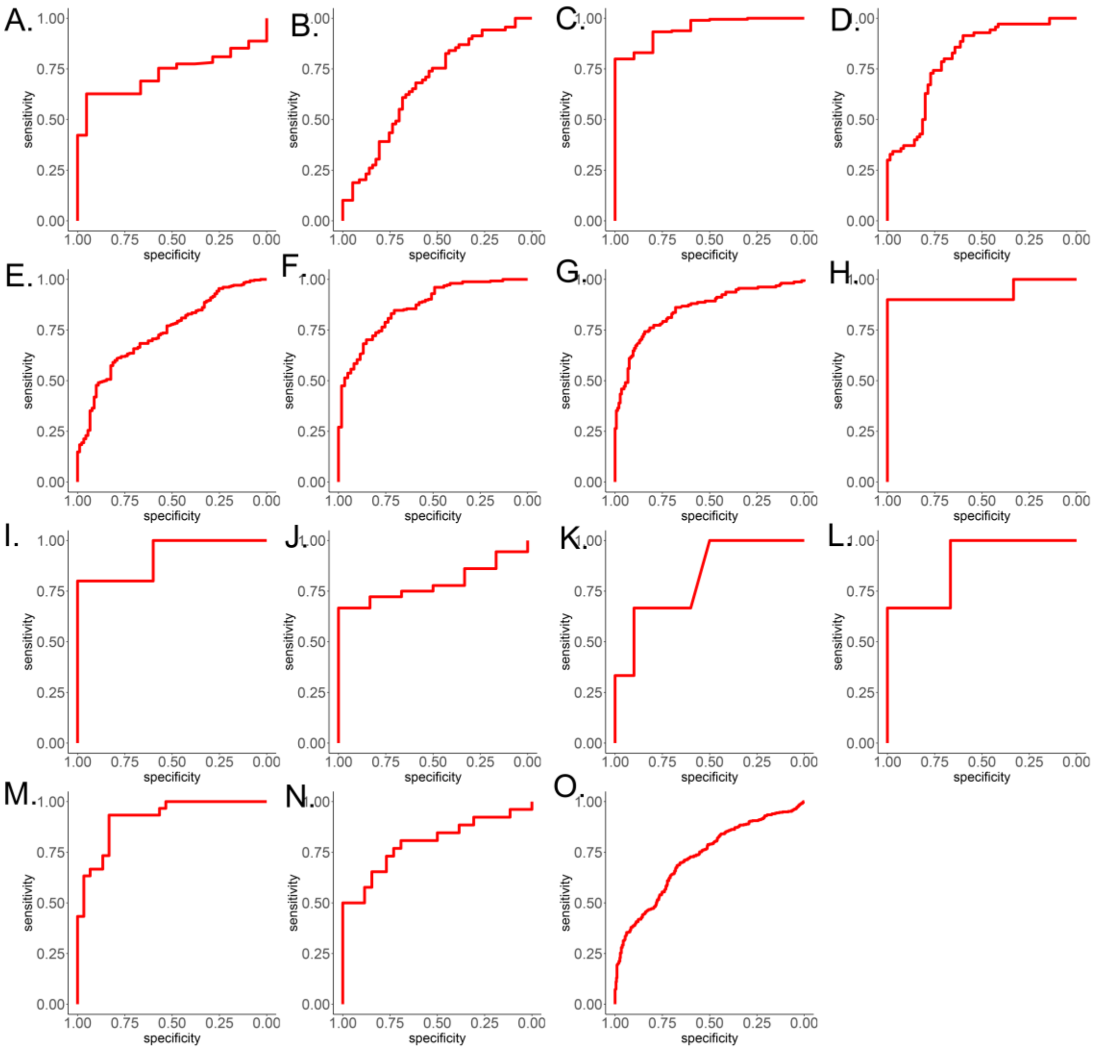

Supplementary Figure 2 Roc curves were performed for each study, AUC was also marked out to access the diagnostic significance.

(A): ArrayExpress_Affaymetrix (B): GPL17077 (C): GPL8573 (D): GPL5175 (E): GPL570 (F): GPL6947 (G): GPL96 (H): GSE103236 (I): GSE109476 (J): GSE112369 (K): GSE116312 (L): GSE158662 (M): GSE30727 (N): GSE51575 (O): TCGA_GTEx

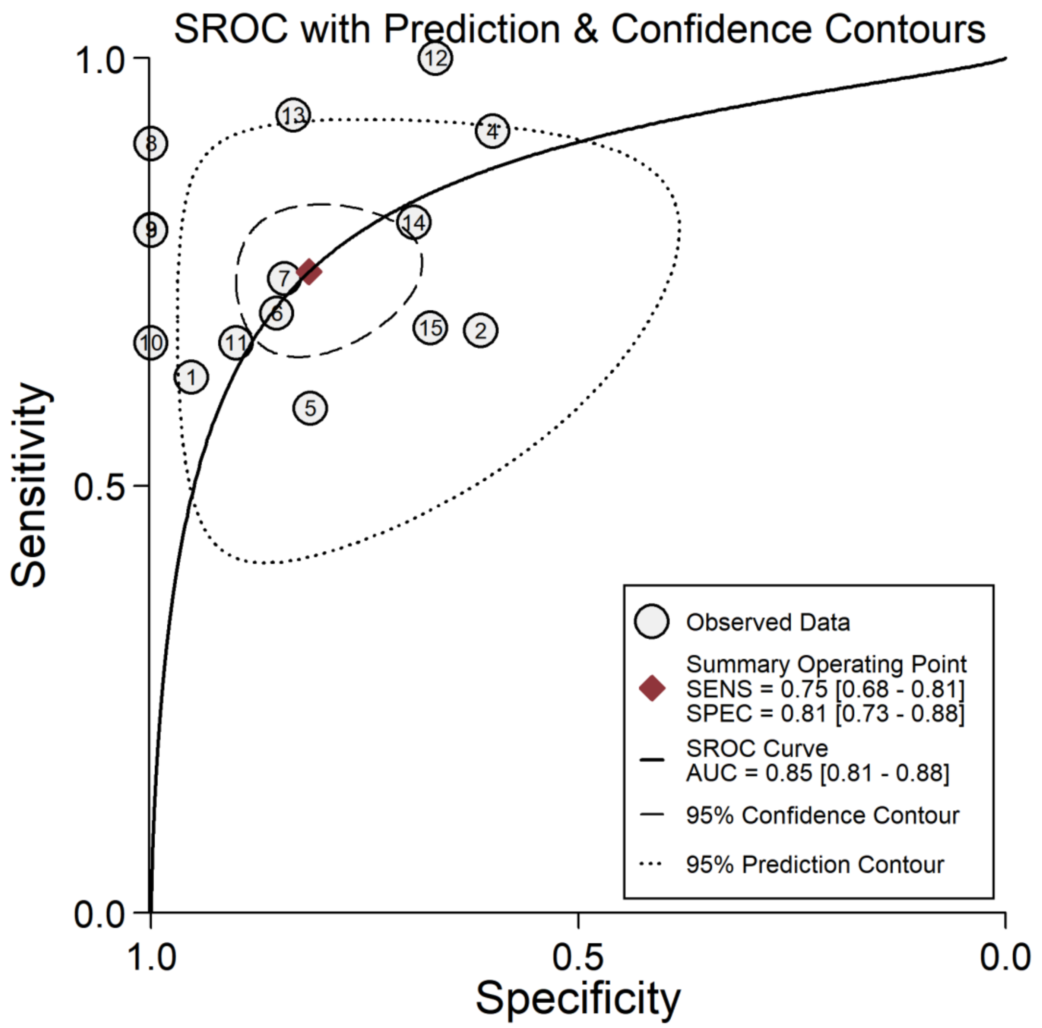

Supplementary Figure 3 SROC curves and was drawn for integrated studies based on tp, fp, fn, tn. (A): The AUC of SROC curve was 0.85 (95%CI=0.81－0.88) with a sensitivity=0.75 (95%CI=0.68－0.81) and a specificity=0.81 (95%CI=0.73－0.88).

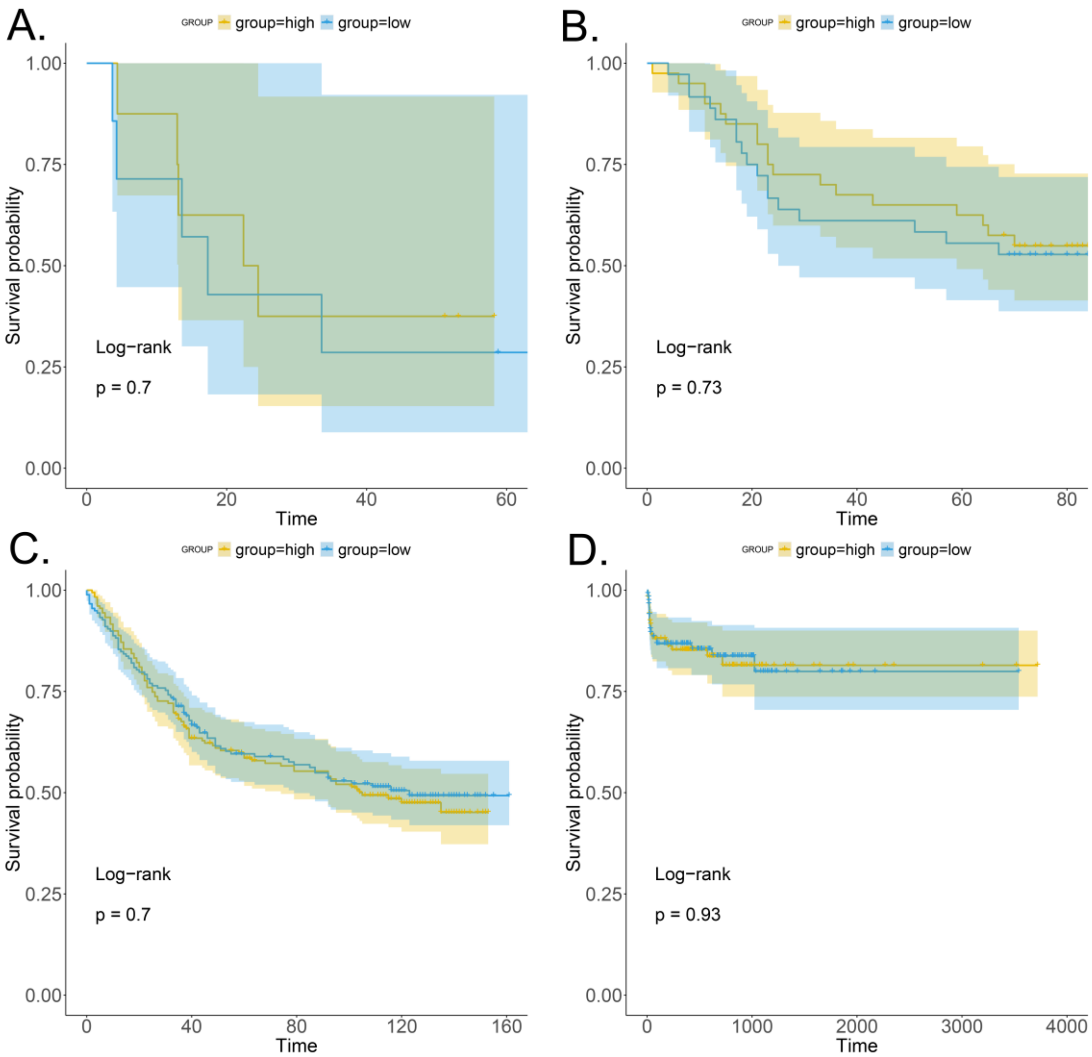

Supplementary Figure 4 The prognostic analysis of LPCAT1. The patients were evenly divided into two groups according to the expression of LPCAT1. High-expressed group was used yellow to indicate and low-expressed group was used blue. LPCAT1 had no prognostic value in GC patients.

(A-D): datasets of GSE38749, GSE84426, TCGA and GSE84433.

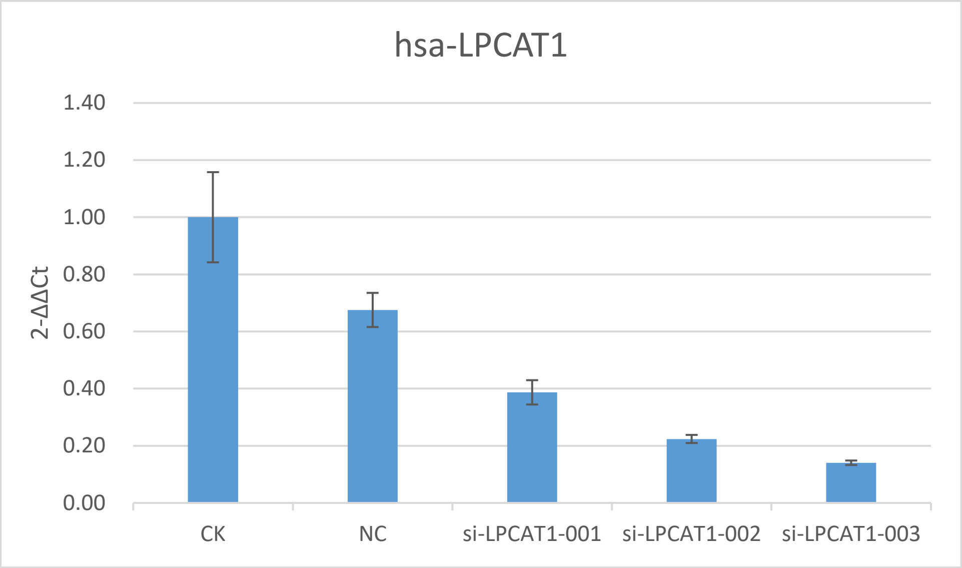

Supplementary Figure 5 The expression of LPCAT1 in CK group, NC group and transfection group. Total three siRNA of LPCAT1 from RIBOBIO were used and si-LPCAT1-003 was the best siRNA.

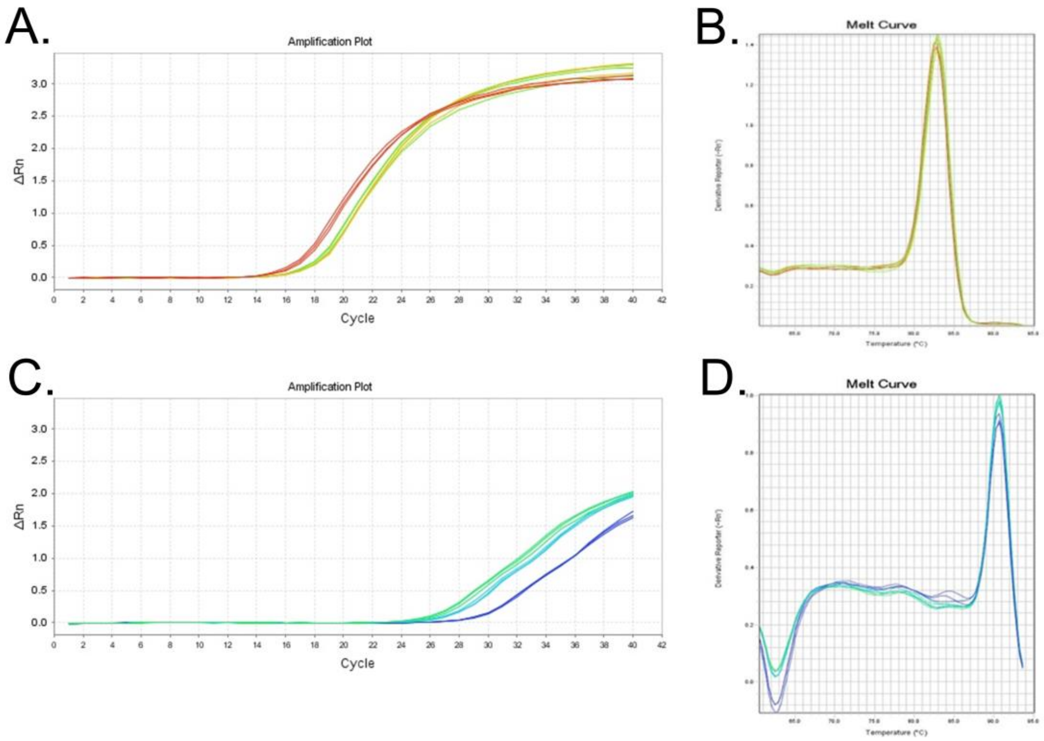

Supplementary Figure 6 The amplification curves and the dissolution curves of GAPDH and LPCAT1.

(A-B): GAPDH. (C-D): LPCAT1.
